# How to Expand and Fill the Self in Organizations: The Role of Interpersonal Processes in the Employee Organizational Identity Construction

**DOI:** 10.3389/fpsyg.2021.634691

**Published:** 2021-11-25

**Authors:** Junping Sun, Yu Song, Guangtao Yu

**Affiliations:** ^1^Department of Human Resource Management, Business School, Central University of Finance and Economics, Beijing, China; ^2^Department of Business Administration, School of Economics and Management, Southeast University, Nanjing, China

**Keywords:** employee organizational identity, relationship-building, connected self, social comparison, social identity theory, interpersonal processes

## Abstract

In the era of Volatility, Uncertainty, Complexity, and Ambiguity (VUCA), the fluidity of organizations and the variability of individual work gradually replace the traditional stability and continuity. The question of how to connect employees and organizations has long intrigued researchers and practitioners. Employee organizational identity is the stable force that binds employees to organizations. Drawing on social identity theory, we argue the role of interpersonal processes in the employee organizational identity construction. We suggest that an employee’s relationship-building behaviors can promote employee organizational identity through the connected self. The indirect effect is stronger for employees who make more social comparisons because they are more sensitive to social influence. We collected data through questionnaires of 333 employees using a two-wave research design in China. The results indicate that an employee’s relationship-building behaviors enhance employee organizational identity. The connected self fully mediates the positive relationship between relationship-building and employee organizational identity. The outcomes also show that the positive effect of relationship-building toward connected self is intensified, when an employee engages in more social comparisons. The findings imply that interpersonal processes play an important role in the employee organizational identity construction. Then, the theoretical and practical implications are discussed.

## Introduction

The modern business has entered the era referred to as “VUCA” – an acronym for Volatility, Uncertainty, Complexity, and Ambiguity, which is repeatedly used to describe the turbulent business environment (Baran and Woznyj, 2021). This brings great challenges to organizations and individuals. The VUCA environment undermines some premises of organizational strategies and makes employees become insecure workers filled with uncertainty and anxiety (Petriglieri et al., 2019). An organization, as the Ship of Theseus, in which each plank is seamlessly connected to form the whole, can survive from turbulent commercial storms only if its employees are connected to each other firmly and tightly like the planks (Cannon and Kreutzer, 2018). Meanwhile, the organization is a common life area and an important source of meaning and self-definition for each employee (Ashforth and Mael, 1989; Arne, 2008), and identity has the potential to provide stability and meanings for employees in such a changing and often turbulent social environment (Roodt et al., 2015). Hence, the organization scholars believed that employee organizational identity (EOI) is a stabilizing force that connects individuals to organizations (Ng, 2015; Li and Zhang, 2020), through which the employees feel sense of belonging and the organizations unite the strength of their members.

As identity is among the most popular topics in contemporary organization studies, which is both the basis of understanding individual behaviors and the core of understanding organizational processes (Brown, 2001, 2015; Ybema et al., 2009; Lok, 2010; Miscenko and Day, 2016). Some researchers have begun to explore the formation mechanisms of EOI and suggested that the construction of EOI is an interactive process (Ashforth and Schinoff, 2016). Previous studies have mostly discussed the construction of EOI by examining the interaction between employees and organizations at the subjective or intrapsychic level (Beech, 2008; Gioia et al., 2010). However, as “the people make the place” (Schneider, 1987), the role of interpersonal interactions in the construction of EOI has been neglected. Therefore, this study uses the social identity theory to explore whether and how the interpersonal interactions influence employees’ organizational identities.

Relationships are the threads in the fabric of organizational life (Ehrhardt and Ragins, 2019). In line with this metaphor, organization scholars have recognized that workplace relationships can provide a foundation for organizational attachment (Chiaburu and Harrison, 2008). Relationship-building refers to behaviors of employees that are directed toward initiating social interactions in the work environment (Wanberg and Kammeyer-Mueller, 2000). Employees develop their workplace relationships by interacting with their coworkers, supervisors, and subordinates who act as a bridge between employees and the organization (Ramarajan and Reid, 2020), and the connections to others play a key role in the construction of EOI (Petriglieri et al., 2019). Thus, we propose that relationship-building behaviors would help employees to connect with others and construct their organizational identities.

As yet, we know little about the mechanisms through which relationships attach employees to the organization (Pratt, 2000; Sias, 2009). The identity is dynamic and individuals progress from one construction of self toward another that is typically construed as improvement, growth, or progress in some way (Dutton et al., 2010). Hence, self-concepts of employees changed gradually in the construction of EOI (Onorato and Turner, 2004). Drawing on social identity theory, social identity construction involves the incorporation of the referent person’s attributes or the group’s norms into an individual’s self-concept (Tajfel and Turner, 1986). Employees who engage in relationship-building behaviors are able to interact closely with others, which increases the likelihood that they incorporate their colleagues’ attributes and group’s norms into self-concepts. Relationship-building behaviors can also help employees to develop close relationships with colleagues and construct their selves as connected (Ramarajan and Reid, 2020), and the high-quality work relationships further become the powerful sources of connection, engagement, and vitality (Ehrhardt and Ragins, 2019). The interpersonal interactions with others are fundamental to employees’ emerging self-definitions (Ramarajan and Reid, 2020), and the extent to which the employees will ultimately identify with the organization depends on their relationships with colleagues around them (Pratt, 2000; Dutton et al., 2010). Therefore, we believe that relationship-building behaviors will lead to changes of self-concepts and propose the connected self as a mediating mechanism of the positive relationship between relationship-building and EOI.

**Figure 1:**
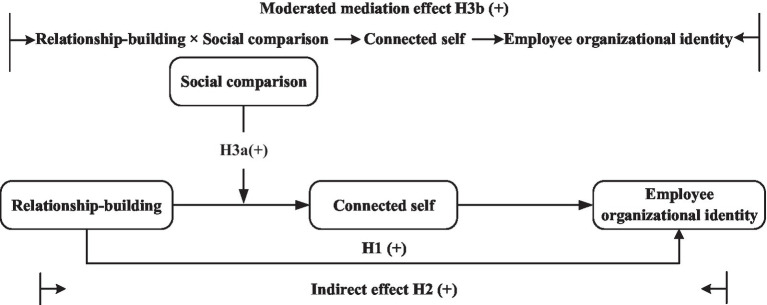
The research model.

Social identity theory indicates that there are two processes in the construction of EOI. One is the social categorization process that produces prototype-based depersonalization of self and generates EOI (Turner et al., 1987; Hogg and Terry, 2000; Hogg and Reid, 2006; Hogg et al., 2017). The other is the social comparison process that provides information about the group prototype and promotes the construction of EOI (Hogg, 2000a). According to social identity theory, social categorization and social comparison are mutually dependent and complementary processes that are indispensable in the construction of social identity (Hogg, 2000b). However, there is little systematic attention paid by social identity theorists to the social comparison process (Hogg, 2000a). In order to fully explore the role of interpersonal processes in the construction of EOI, the researchers should consider the effects of social categorization and social comparison at the same time. As social categorization is relevant to social interaction (Hogg, 2000a), we regard relationship-building behaviors as a means of social categorization. Additionally, along with the employees’ interpersonal interactions with their colleagues, intra-organization social comparisons that drive employees toward conformity will occur naturally (Hogg, 2000a; Bartel, 2001). Thus, we propose that social comparisons facilitate an employee’s construction of the connected self when he or she engages in relationship-building behaviors. That is, social comparisons moderate the positive relationship between relationship-building and connected self. Our theoretical model is illustrated in Figure 1.

This study contributes to literatures of the EOI and social identity theory in three ways. First, this study expands social identity theory by combining the social categorization process with the social comparison process. Both are important to the construction of EOI (Bartel, 2001), and the facilitation effect of social comparisons on the social categorization process is discussed thoroughly. Second, this study extends the research about EOI by examining the effects of relationship-building behaviors and social comparison behaviors on EOI at the same time, which reveals the key role of interpersonal processes in EOI construction. Finally, this study extends the research on the expansion of self-concept. The connected self is introduced firstly as a mediating variable to explain the influencing mechanism of interpersonal processes in the EOI construction, and the EOI can be seen as an expanded and filled self within the organization.

## Theory and Hypotheses

Social identity theory is a social psychological analysis, which reveals the role of self-concept in group members, group processes, and inter-group relations (Tajfel and Turner, 1979). It focuses on the social cognition, motivation, social interaction, and macro social aspects of the group life. Turner (1982) further elaborated the process of social influence within a group under social identity theory, which is called as referent informational influence. In other words, people build group norms from behaviors of group members and internalize these norms as part of their social identities. The core component of social identity theory is the formation of the self-concept (Tajfel and Turner, 1986).

There are two types of self-concepts. One is the personal self and the other is the social self. There are two levels of social selves. The relational self is the self-concept derived from connections and interpersonal relationships with specific others. The collective self is the self-concept derived from membership in larger, more impersonal collectives or social categories (Brewer and Gardner, 1996). The collective self is corresponding to the concept of social identity as represented in social identity theory (Tajfel, 1982; Turner et al., 1987). Tajfel (1978, p.63) defined social identity as “that part of an individual’s self-concept which derives from his knowledge of his membership of a social group together with the value and emotional significance attached to that membership.” Previous researchers have advocated for transforming the definition of “social identity” into “collective identity” (Brewer and Gardner, 1996; Sedikides and Brewer, 2001; Simon and Klandermans, 2001). An organization is a collective (Hannan, 2005). Therefore, based on the definition of social identity provided by the social identity theory (Tajfel, 1978), we define EOI as that part of an employee’s self-concept that derives from his or her knowledge of his or her membership of an organization together with the value and emotional significance attached to that membership.

Social identity theory attaches employees to the organization by the means of self-concept, and self is the articulatory mechanism between employees and the organization. Employees and organizations are fused within the collective self (Hogg and Williams, 2000). The construction of EOI is essentially a process that employees define themselves in terms of organizational attributes (Ashforth and Mael, 1989), which is “to a very large extent relational and comparative” (Tajfel and Turner, 1986; Zhang et al., 2020a). Social identity theory suggests the prototype is a cognitive abstraction of the central features of the membership category. It is an exemplar of the category to which employees compare themselves (Burke and Stets, 2000). Identity construction processes involve individuals’ taking on or modifying some aspect of a self-concept (Dutton et al., 2010).

### Relationship-Building and Employee Organizational Identity

Identity is formed in practice (Lerpold et al., 2007). Social identity theory proposes that EOI has focused on common or shared outcomes for organizational members (Burke and Stets, 2000). According to social identity theory, EOI is socially constructed (Glynn and Navis, 2013) and related to social interactions (Postmes et al., 2005; Hogg and Reid, 2006). Relationship-building is an important tactic during the organizational entry (Ashford and Black, 1996) and emphasizes on the social relational aspect of the entry (Wang et al., 2015). Specifically, this tactic is aimed at seeking out interaction opportunities, which can give employees friendship networks, social support, and situational identities (Morrison, 1993). Employees construct their organizational identities based on work friendships and daily work interactions (Berman et al., 2002). Relationship-building improves employees’ social resources that are closely related to the positive identity construction (Dutton et al., 2010) and help them to integrate themselves into the organization (Wanberg and Kammeyer-Mueller, 2000). Thus, we hypothesize the following:

***Hypothesis 1 (H1):*** Relationship-building is likely to lead to increased EOI.

### Mediating Role of the Connected Self

Connected self means that employees construct their selves as “connected”: in a close relationship with colleagues that is characterized by empathy, mutual understanding, and respect (Ramarajan and Reid, 2020). As a kind of self-construction, the connected self also implies that people define themselves by their relationships with others (Sparrowe, 2005; Ramarajan and Reid, 2020). Carlsen (2006) depicted how individuals alter self-constructions at work in ways that enable seeing themselves as progressing in their overall life narratives. Social identity theory argues that self-concept is highly variable and context-dependent (Turner et al., 1994; Onorato and Turner, 2004). Turner (1982) proposed that an individual’s self-concept could itself be defined along a continuum ranging from a definition of the self in terms of personal identity to a definition in terms of social identity. The shift from personal identity to social identity represents the fluidity in the self-concept (Onorato and Turner, 2004). Individuals have a fundamental need to feel a sense of interpersonal belonging with others around them (Cooper et al., 2020). They meet this need by expanding their self-concepts to include connections with others (Baumeister and Leary, 1995; Ashforth et al., 2008).

According to social identity theory, the construction of EOI emphasizes interpersonal relationships, which forms part of the employee’s self-concept or identity (Stryker and Serpe, 1982; Deaux and Martin, 2003). Hence, the quality of EOI depends on the extent to which relationships change the self-concept (Pratt, 2000). Individuals generally strive to maintain agreement, develop mutually acceptable selves, and attempt to socially validate those selves through interactions (Ashforth, 2001). Previous studies have demonstrated that greater contacts, identifying similarities, and practicing self-disclosure can result in closer relationships with others (Miller, 2002; Davies et al., 2011). Relationship-building behaviors reinforce friendship networks and social support (Nelson and Quick, 1991). Employees take active steps to expand their social networks, by initiating conversations with their colleagues and getting to know them better (Tan et al., 2016). These will improve their chances of being accepted by organizational members. With the increase of relationship-building behaviors, employees will constantly incorporate interactions between colleagues and themselves into their self-concepts (Linardatos and Lydon, 2011). Employees construct their connected selves through drawing information that they had gleaned about their colleagues together with experiences of moments of closeness (Ramarajan and Reid, 2020). Therefore, relationship-building behaviors of employees promote the formations of their connected selves.

Employees with connected selves are in close relationships with their colleagues. Over time, the positive evaluations of relationships will strengthen the construction of EOI through affect transfer, behavioral sensemaking, and social influence (Sluss et al., 2012). Deaux and Martin (2003) further clarified that social identities are enacted through the interpersonal networks of daily life. These networks consist of colleagues who share the organizational membership. Accumulations of moments over time and across colleagues provided the employee with the raw materials – shared experiences, strong emotions, and new information about his or her own and colleagues’ social and personal backgrounds – that informed how the employee constructed his or her organizational identity (Ramarajan and Reid, 2020). According to social identity theory, the construction of EOI is a process of learning shared organizational prototypes and norms, which is related to social interactions. Actions and words of employees convey relevant information about prototypes and norms (Hogg and Reid, 2006). Employees with connected selves can obtain more information about organizational norms and prototypes from colleagues. They further change their self-concepts and assimilate their own attitudes, feelings, and behaviors into an organizational prototype (Hogg, 2001). At the same time, employees with connected selves are more likely to have something in common with organizational members. Attachment/belonging/closeness is a central factor in identity construction (Knez, 2016). Thus, we hypothesize the following:

***Hypothesis 2 (H***_***2***_***):*** The connected self is likely to mediate the positive relationship between relationship-building and EOI.

### Moderating Effects of Social Comparison Orientation

All interactions have identity effects but more specific processes regulate their actual impacts (Karreman and Alvesson, 2001). The existing literatures on the social identity theory posited that the social identity construction depends on social categorization and social comparison processes (Hogg, 2000b). Social comparison process is the value foundation of social identity construction (Deaux and Martin, 2003). Social comparisons are important means for employees to construct their organizational identities (Bartel, 2001). Individuals compare themselves with others according to their social category, which is accompanied by emotion, evaluation, and certain behaviors (Van Dick et al., 2004). Intra-organization social comparisons with colleagues promote similarity, assimilation, and consistency among individuals (Turner, 1975; Bartel, 2001). The purpose of intra-organization social comparisons is to accurately establish the group prototype and assimilate the self to the prototype (Hogg, 2000a). Therefore, we propose that intra-organization social comparison will be an important boundary condition, which influences the effects of interpersonal processes on EOI construction.

Individuals make social comparisons to determine that one’s opinions are correct and precisely what one is and is not capable of doing (Festinger, 1954). There is a “unidirectional drive upward” that drives individuals to compare themselves with someone slightly better than themselves. Previous studies have shown that interpersonal upward comparisons can satisfy individuals’ self-improvement motivation (Major et al., 1991). Hence, intra-organization comparisons are more likely to be upward comparisons rather than downward comparisons. Within an organization, employees are more likely to make comparisons with prototypical members based on organizational membership (i.e., upward comparisons). Employees can directly obtain information about the organizational prototype and prototype their behaviors from these comparisons (Hogg, 2000a).

On the basis of these arguments, we propose that organizational members who make more social comparisons are able to more accurately discern and describe the appropriate behaviors and attitudes related to interactions with coworkers. Employees who make fewer social comparisons cannot determine the relevant information about organizational prototypes and norms that are conveyed by employees’ actions and words (Hogg and Reid, 2006). They do not know how to make themselves more consistent with other organizational members. Their relationship-building behaviors cannot forge connections with other organizational members. Hence, fewer social comparisons with colleagues weaken the positive relationship between relationship-building and the connected self. On the contrary, employees who make more social comparisons are able to discover the differences between themselves and the prototypical members. They learn from the prototypical members and make adjustments in the process of relationship-building to promote their connected selves. Hence, more social comparisons with colleagues will enhance the positive relationship between relationship-building and the connected self. Furthermore, relationship-building promotes the connected self and thus increases the construction of EOI. Therefore, social comparisons also enhance the mediated relationship between relationship-building and EOI through the connected self. Thus, combined with H_2_, we propose the following hypothesis:

***Hypothesis 3a (H***_***3a***_***):*** Social comparison is likely to moderate the positive relationship between relationship-building and the connected self. That is, the positive relationship between relationship-building and the connected self is stronger for employees who make more social comparisons rather than those who make fewer social comparisons.

***Hypothesis 3b (H***_***3b***_***):*** Social comparison is likely to moderate the mediated relationship between relationship-building and EOI through the connected self. That is, the mediating effect is stronger for employees who make more social comparisons rather than those who make fewer social comparisons.

## Materials and Methods

### Design and Participants

We used the online survey platform, Credamo, to collect data from ordinary staff from all walks of life and managers at all levels of enterprises in China. In order to better verify the research model, we limited the occupations of the samples on the questionnaire survey platform. We only issue questionnaires for managers (including junior and senior managers), general staff (office workers) and professional staff (such as doctors, lawyers, journalists, and teachers). Compared with other occupations (such as students, laborers, government, agricultural, forestry, animal husbandry, fishery workers, etc.), the working environment to these people is more in line with general organizational situation. At the same time, in order to ensure the quality of the questionnaire survey, we selected the subjects on the platform whose credit scores are greater than or equal to 60.^1^ We allowed all participants to decide at any time whether to continue participating in the survey. We added a specified option question in the first phase of the questionnaire design so that the platform could automatically reject participants who did not examine the questions and options at all to ensure the validity of the replies (Huang et al., 2012; Desimone et al., 2015). We offered a reward of 6 yuan for each questionnaire to arouse participant enthusiasm (Zhang et al., 2020b).

To reduce common method biases (CMBs), we adopted a procedural design suggested by Podsakoff et al. (2003) to control for common method variance. First, in the questionnaire’s guidance language, we emphasized that the results of the survey would only be used for the academic research, that no answer is right or wrong, and that all the participants’ information would be kept strictly confidential. Second, we collected longitudinal data through a phased investigation. In the first stage, we collected the employees’ relationship-building behaviors, connected selves, social comparison behaviors, demographic information, and the collectivism orientation of the employees. We issued 450 questionnaires and received 401 responses, representing an 89.11% response rate. In the second stage, we collected the organizational identities of the employees, issued 401 questionnaires, and received 388 responses, representing 96.76% response rate. Each stage was conducted 2weeks apart. After deleting the questionnaires in which a participant took less than 2s to answer each question (exhibiting an obvious ineffective response tendency) and outliers (Huang et al., 2012), we obtained a total of 333 valid questionnaires as the final sample, with a total effective response rate of 74%. The mean age of the participants was 30.294 (SD=4.682). The average work experience of the participants in their current organization was 5.880years (SD=4.059), of which 57.96% were 1–5years, 31.53% were 6–10years, and 10.51% were 11–25years. In terms of gender, there were 161 males, accounting for 48.35% of the total sample. In terms of educational background, 12.31% of the participants reported having a college degree or below and 81.08% reported having a bachelor’s degree, and 6.61% reported having a master’s degree. In terms of position, 33.03% were general staff, 44.45% were junior managers, 21.62% were middle managers, and 0.90% were senior managers. Most of the employees (77.48%) were general employees or junior managers.

### Variables and Questionnaires

The present study used two questionnaires to investigate employees. The Phase I questionnaire contained demographic variables and a scale measuring employees’ collectivism orientation, relationship-building behaviors, connected selves, and social comparison behaviors. The Phase II questionnaire included a scale assessing EOI. All questionnaire items were filled out by the employees.

We adopted the maturity scales previously published by scholars and made adjustments to fit the present study. All measures were translated into Chinese using a standard back-translation procedure (Brislin, 1986). We invited four doctoral students and one management professor of our major to compare and revise the translation of the measurement items and repeatedly compared the original scale with the translated scale.

#### Relationship-Building

Relationship-building was measured using a nine-item scale designed by Ashford and Black (1996). This scale measures three dimensions: general socializing, networking, and boss, with each dimension containing three items. Sample items are “Participated in social office events to meet people (i.e., parties, softball team, outings, clubs, and lunches)” (general socializing), “Started conversations with people from different segments of the company” (networking), and “Tried to spend as much times as you could with your boss” (boss). The questionnaire used a five-point Likert scale (1=“very infrequently” and 5=“very frequently”). The Cronbach’s alpha for this measure was 0.891.

#### Connected Self

There is no scale that directly measures the connected self (Ramarajan and Reid, 2020). We used the social learning outcome as a measure of the connected self for the following three reasons. First, by definition, both emphasize the strength of social relationships with colleagues. The social learning outcome refers to getting to know colleagues and being liked and accepted by them (Wanberg and Kammeyer-Mueller, 2000; Bauer et al., 2007; Tan et al., 2016). Second, in the development process of employees in the organization, both belong to the adjustment stage, which means that they are not the final result of identity construction, but rather an ongoing process (Collin, 2009; Ramarajan and Reid, 2020). Finally, in terms of variable measurement, both are applicable to self-reporting. The reason why we used self-reporting to measure the connected self is that categorization is derived from the social perceiver’s evaluation of his or her own relationships with others, rather than from the perspective of others (Burke and Stets, 2000).

Therefore, we used the social learning outcome scale used by Tan et al. (2016) to measure the connected self, with a total of six items. Two sample items are “My relationships with other workers in this company are very good” and “I am usually included in informal networks or gatherings of people within this organization.” The questionnaire adopted a seven-point Likert scale (1=“strongly disagree,” 7=“strongly agree”). The Cronbach’s alpha for this measure was 0.869.

#### Social Comparison

We used the social comparison orientation scale developed by Gibbons and Buunk (1999) to measure social comparison behaviors of employees, with a total of five items. Two sample items are “I pay a lot of attention to how I do things compared with how others do things” and “I compare how I am doing socially (e.g., social skills and popularity) with other people.” The questionnaire used a five-point Likert scale (1=“very infrequently” and 5=“very frequently”). The Cronbach’s alpha for this measure was 0.797.

#### Employee Organizational Identity

We used the scale developed by Heere and James (2007) according to the theoretical model of Ashmore et al. (2004) to measure the EOI. The scale measures six dimensions – private evaluation, public evaluation, interconnection of self, sense of interdependence, behavioral involvement, and cognitive awareness – with a total of 21 items. Sample items are “I am proud to think of myself as a member of my organization” (private evaluation); “Overall, people hold a favorable opinion about my organization” (public evaluation); “In general, being associated with the organization is an important part of my self-image” (interconnection of self); “My destiny is tied to the destiny of the organization” (sense of interdependence); “I am actively involved in organization-related activities” (behavioral involvement); and “I am aware of the tradition and history of my organization” (cognitive awareness). The questionnaire adopted a seven-point Likert scale (1=“strongly disagree” and 7=“strongly agree”). The Cronbach’s alpha for this measure was 0.924.

#### Control Variables

We controlled for individual characteristics (i.e., gender, age, educational level, work years, and position) and one organizational characteristic (i.e., organization type) that were expected to be correlated with the connected self and EOI and that might confound our hypothesized relationships. Social identity theory suggests that individual self-concept orientation is the key individual characteristic that affects the construction of EOI (Cooper and Thatcher, 2010). Collectivism/individualism is one of the main dimensions to distinguish Chinese and Western social culture (Hofstede, 2001). Collectivism means that individuals regard themselves as a member of one or more groups and depend on each other (Triandis, 1995, 2001). Combined with the sample for this study, we believe that the collectivism orientation of Chinese individuals will affect the constructions of their collective identities. Hence, we also controlled for the collectivism orientation of employees. The scale was developed by Triandis and Gelfand (1998). The Cronbach’s alpha for this measure was 0.862.

#### Statistical Analysis

The data analysis was conducted in three steps using SPSS version 26.0 and Mplus version 7.4 software. First, a series of confirmatory factor analyses (CFA) was performed using Mplus 7.4 software to test the discriminant validity of the variables.

Second, we tested for possible common method variance using the Harman’s single-factor test and the unmeasured latent methods factor technique (Podsakoff et al., 2003).

Third, we tested all the research hypotheses using SPSS 26.0 software, applying the PROCESS macro (version 3.4). The assumptions for the calculation of the regression analysis have been checked and found to be satisfactory. The results of Durbin-Watson Statistic showed that all DWs were close to 2, so the independent variables had no autocorrelation. The Variance Inflation Factor (VIF) of each independent variable was less than two, which means that there was no perfect multicollinearity among the explanatory variables. In order to overcome the issue of non-normal sample distribution and more accurately examine the indirect effects, we used the bias-corrected bootstrapping method with 5,000 resamples to test the mediating effect and the moderated mediation effect.

## Results

### Confirmatory Factor Analyses

We conducted CFA using Mplus 7.4 software to test the discriminant validity of the variables. We compared the goodness-of-fit indices of the four-factor model with other nested models. As shown in Table 1, the CFA results showed that the hypothesized four-factor model (*χ*^2^=604.591, *df*=288, *χ*^2^/df=2.099, RMSEA=0.057, CFI=0.937, TLI=0.927, SRMR=0.054) fitted the data better than the three-factor model 1 (*χ*^2^=1572.294, *df*=296, *χ*^2^/*df*=5.312, RMSEA=0.114, CFI=0.721, TLI=0.693, SRMR=0.091), the three-factor model 2 (*χ^2^*=1401.200, *df*=296, *χ*^2^/df=4.734, RMSEA=0.106, CFI=0.758, TLI=0.735, SRMR=0.081), the two-factor model (*χ*^2^=1921.891, *df*=298, *χ*^2^/*df*=6.449, RMSEA=0.128, CFI=0.645, TLI=0.613, SRMR=0.107), or the one-factor model (*χ*^2^=2509.030, *df*=299, *χ*^2^/*df*=8.391, RMSEA=0.149, CFI=0.517, TLI=0.475, SRMR=0.123), which indicated that the four constructs captured distinctiveness as expected in this study.

**Table 1:** Results of confirmatory factor analysis.

Model	*χ* ^2^	*df*	*χ*^2^/*df*	RMSEA	CFI	TLI	SRMR
Four-factor model	604.591	288	2.099	0.057	0.931	0.922	0.054
Three-factor model 1	1572.294	296	5.312	0.114	0.721	0.693	0.091
Three-factor model 2	1401.200	296	4.734	0.106	0.758	0.735	0.081
Two-factor model	1921.891	298	6.449	0.128	0.645	0.613	0.107
One-factor model	2509.030	299	8.391	0.149	0.517	0.475	0.123

*N=333, Four-factor model: Relationship-building, connected self, social comparison, EOI; Three-factor model 1: Relationship-building+connected self, social comparison, EOI; Three-factor model 2: Relationship-building+social comparison, connected self, EOI; Two-factor model: Relationship-building+connected self+social comparison, EOI; One-factor model: Relationship-building+connected self+social comparison+EOI*.

### CMB Testing

Although procedural remedies have been adopted in this study to reduce the impact of common method variance, all the variables in this study were reported by employees, so common method variance might still exist. There were two techniques to test the seriousness of any common method variance (Podsakoff et al., 2003). First, Harman’s single-factor test results showed that the variance interpretation rate percentage of the first common factor was 29.683%, less than 40%, and the fit of the one-factor model was very poor (*χ*^2^=2509.030, *df*=299, *χ*^2^/*df*=8.391, RMSEA=0.149, CFI=0.517, TLI=0.475, SRMR=0.123). Second, the five-factor model with common method factors and data could not be fit. Compared to the fit of the four-factor model, the goodness-of-fit indices of the latent method factor model did not improve. These results indicated that common method variance was not a serious problem.

### Descriptive Statistics and Correlations

The means, standard deviations, scale reliabilities of variables, and the matrix of Pearson correlations among the constructs are presented in Table 2. The Cronbach’s *α* of each variable was greater than 0.70, indicating good reliability. The correlations indicated that relationship-building was positively related to EOI (*r*=0.358, *p*<0.001) and the connected self (*r*=0.543, *p*<0.001) and that the connected self was positively related to EOI (*r*=0.571, *p*<0.001). These results offered preliminary support for our theoretical hypotheses.

**Table 2:** Means, standard deviations, scale reliabilities, and Pearson’s correlations among constructs.

Variables	*M*	*SD*	1	2	3	4	5	6	7	8	9	10	11
1. Gender	0.517	—	—										
2. Age	30.294	4.682	−0.061	—									
3. Education	2.934	0.461	−0.047	−0.163^**^	—								
4. Organizational tenure	5.880	4.059	−0.015	0.796^***^	−0.140^*^	—							
5. Position	1.904	0.758	−0.210^***^	0.226^***^	0.111^*^	0.254^***^	—						
6. Organization type	1.883	0.826	0.016	−0.047	−0.147^**^	−0.101	−0.028	—					
7. Collectivism orientation	5.453	0.904	0.103^†^	0.162^**^	−0.004	0.147^**^	0.160^**^	−0.031	(0.862)				
8. Relationship-building	3.552	0.708	0.033	0.024	0.045	0.028	0.165^**^	0.059	0.485^***^	(0.891)			
9. Social comparison	3.480	0.726	−0.084	0.073	0.099	0.087	0.005	−0.015	0.158^**^	0.399^***^	(0.797)		
10.Connected self	5.652	0.766	0.048	0.055	0.085	0.097	0.135^**^	−0.038	0.591^***^	0.543^***^	0.185^**^	(0.869)	
11.Employee organizational identity	5.718	0.571	0.061	0.180^**^	−0.049	0.188^**^	0.154^**^	−0.059	0.477^***^	0.358^***^	0.178^**^	0.571^***^	(0.924)

*N=333. Gender is coded 0 for male and 1 for female. Age and Organizational tenure are continuous variables that are the actual number of years. Education is an ordered variable, 1=high school and below, 2=junior college, 3=undergraduate, 4=postgraduate, 5=doctoral. Position is an ordered variable, 1=general staff, 2=grass-roots managers, 3=middle-level managers, and 4=senior managers. Organization type is a categorical variable, 1=state-owned enterprise, 2=private enterprise, 3=Sino-foreign joint venture, and 4=wholly foreign-owned enterprise. The Internal consistency coefficients are reported on the diagonal (two-tailed). ^†^p<0.1*;

* *p<0.05*;

** *p<0.01*;

*** *p<0.001*.

### The Mediating Role of the Connected Self

First, we used hierarchical regression analysis and the bias-corrected bootstrapping method to test the research hypotheses (see Tables 3 and 4). From Model 5 in Table 3, we attained results showing that relationship-building was positively related to EOI (*β*=0.141, *p*=0.002, BC bootstrap 95% CI=[0.0542, 0.2287]), after controlling for demographic variables (i.e., gender, age, educational level, work years, position, organization type, and collectivism orientation). The positive relationship between relationship-building and EOI was significant. This finding provided support for H_1_.

**Table 3:** Results of regression analysis.

Variables	Dependent variables						
	Connected self			Employee organizational identity			
	Model 1	Model 2	Model 3	Model 4	Model 5	Model 6	Model 7
Constant	2.984	3.790	3.864	4.133	4.449	3.193	3.218
**Control variables**							
Gender	−0.015	−0.015	−0.021	−0.034	0.034	0.039	0.048
Age	−0.021^†^	−0.017	−0.016	0.003	0.004	0.010	0.010
Education	0.132^†^	0.111	0.121^†^	−0.056	−0.064	−0.101^†^	−0.110^†^
Organizational tenure	0.022	0.022^†^	0.019	0.011	0.011	0.004	0.002
Position	0.029	−0.005	0.002	0.051	0.038	0.039	0.048
Organization type	−0.002	−0.026	−0.040	−0.029	−0.039	−0.030	−0.030
Collectivism orientation	0.501^***^	0.365^***^	0.335^***^	0.282^***^	0.229^***^	0.108^**^	0.108^**^
**Independent variable**							
Relationship-building		0.361^***^	0.418^***^		0.141^**^	0.022	0.0003
**Mediator**							
Connected self						0.331^***^	0.330^***^
**Moderator**							
Social comparison			−0.048				0.057
**Interaction**							
Relationship-building × Social comparison			0.230^***^				0.022
*R^2^*	0.364	0.447	0.474	0.249	0.272	0.381	0.386
*∆R^2^*		0.083	0.110		0.023	0.132	0.137
F	26.572^***^	32.693^***^	29.033^***^	15.385^**^	15.115^***^	22.116^***^	18.353^***^
DW	1.953	1.995	1.993	1.928	1.932	1.955	1.950

*N=333. Gender is coded 0 for male and 1 for female. Age and organizational tenure are continuous variables that are the actual number of years. Education is an ordered variable, 1=high school and below, 2=junior college, 3=undergraduate, 4=postgraduate, 5=doctoral. Position is an ordered variable, 1=general staff, 2=grass-roots managers, 3=middle-level managers, and 4=senior managers. Organization type is a categorical variable, 1=state-owned enterprise, 2=private enterprise, 3=Sino-foreign joint venture, and 4=wholly foreign-owned enterprise (two-tailed). ^†^p<0.1*;

^*^p<0.05;

** *p<0.01*;

*** *p<0.001*.

**Table 4:** Bootstrapping analysis of the mediation effect of the connected self.

Relationship-building → Connected self → EOI	Effect	SE	LLCI	ULCI
Direct effect	0.022	0.044	−0.0645	0.1082
Indirect effect	0.120	0.027	0.0713	0.1797

Furthermore, the results of Model 2 in Table 3 indicated that relationship-building was positively related to the connected self (*β*=0.361, *p*<0.001, BC bootstrap 95% CI=[0.2588, 0.4629]) and the results of Model 6 in Table 3 indicated that the connected self was positively related to EOI (*β*=0.331, *p*<0.001, BC bootstrap 95% CI=[0.2452, 0.4177]), after controlling for relationship-building and demographic variables (i.e., gender, age, educational level, work years, position, organization type, and collectivism orientation; see Table 3). There was no effect of relationship-building on EOI. According to MacKinnon et al. (2007), the connected self fully mediated the effects of relationship-building on EOI. Bootstrapping analysis with 5,000 bootstrap samples further confirmed the significance of the indirect effect of connected self between relationship-building and EOI (indirect effect=0.120, BC bootstrap 95% CI=[0.0713, 0.1797], excluding 0; see Table 4). These findings provided support for H_2_.

### The Moderating Role of Social Comparison

**Figure 2:**
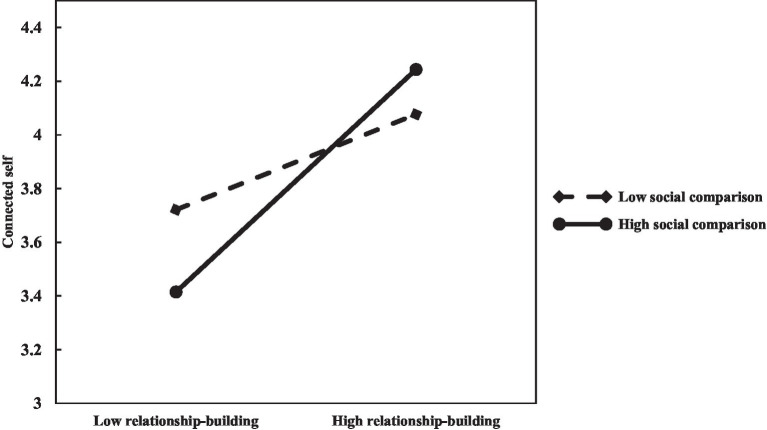
The moderating effect of social comparison on the relationship between relationship building and connected self.

Following Aiken and West (1991), we entered the employees’ gender, age, educational level, work years, position, collectivism orientation, and organization type as control variables in the first block of the regression equation. In the second step, the independent variable (relationship-building) was entered to test for the main effect. In the third step, we entered the moderator variable (social comparison). Finally, the multiplicative interaction term between relationship-building and social comparison was entered in the fourth step. Independent and moderator variables were mean-centered. We tested the multiplicative interaction term in the first stage and found that social comparison significantly moderated the relationship between relationship-building and the connected self (*β*=0.230, *p*<0.001BC bootstrap 95% CI=[0.1181, 0.3429]; see Model 3 in Table 3). The effect of relationship-building on the connected self was significant and amplified by social comparison. Figure 2 illustrated the moderating effect of social comparison on the relationship between relationship-building and the connected self. The low social comparison stood for −1 SD. And the high social comparison stood for +1SD. Thus, H_3a_ was supported.

The index of moderated mediation was presented in Table 5. The index for EOI was 0.076, with a confidence interval (BC bootstrap 95% CI=[0.0299, 0.1305]) excluding zero. Thus, H_3b_ was supported.

**Table 5:** Summary of moderated mediation.

Moderator variable	Model	Index	SE	LLCI	ULCI
Social comparison	Relationship-building → Connected self → EOI	0.076	0.026	0.0299	0.1305

## Discussion

The emerging research on identity construction has drawn the attention of scholars to the interpersonal relationships during the construction of EOI (Cooper et al., 2020). Drawing on social identity theory, we explored and tested the effect of relationship-building on EOI, the underlying mechanism that might explain the effect and the moderation of the effect by employees’ social comparison. We conducted questionnaires of 333 employees based on a two-wave research design. In short, the results of our empirical test demonstrate that the impact of relationship-building on EOI is mediated by employees’ connected self, and the indirect effect is moderated by employees’ social comparison behaviors. Our findings show that the construction of EOI is influenced by relationship-building and social comparison behaviors. And, this process is realized by expanding employees’ self-concepts. These findings carry several implications for research on EOI and social identity theory, as well as practice.

### Theoretical Implications

This research extends our knowledge on the influence of interpersonal processes on EOI and its underlying mechanism and makes contributions to the construction of EOI in three notable ways.

First, this study expands social identity theory by incorporating relationship-building and social comparison as antecedents of EOI. Social identity theory suggests that social categorization and social comparison processes are very important to the identity construction (Abrams and Hogg, 2004; Hogg, 2006; Bothma et al., 2015). Bartel (2001) advised that scholars should simultaneously consider the influence of both social categorization and social comparison processes during the construction. Reid and Hogg (2005) used two group experiments to test the prediction that motivations of social categorization and intergroup social comparison have an interactive effect on ingroup identity. But they did not explore the specific behaviors in the process. We regarded relationship-building as behaviors of social categorization process and considered intra-organization social comparison with colleagues as behaviors of social comparison process. As a response to the call from Bartel (2001) and drawing on findings of Reid and Hogg, we tested the interactive effect of relationship-building and social comparison during the identity construction. Thus, we gained a more integrated and specific understanding of social identity theory.

Second, this study also expands the research on EOI by focusing on interpersonal interactions. Existing studies have emphasized the changes at a subjective or intrapsychic level during the construction of EOI (e.g., cognitive identity work, self-reinforcing mechanism, defense, and coping mechanisms; Löwstedt and Räisänen, 2014; Petriglieri, 2015; Caza et al., 2018; Frey, 2020), while the impact of interpersonal interactions on EOI is largely unexplored. Ramarajan and Reid (2020) used a qualitative, theory-building approach to describe how agents engaged in a typically series of interpersonal interactions that reshaped their self-concepts. Based on this research, we used a quantitative approach to investigate the influence of interpersonal interactions (i.e., relationship building and social comparison behaviors) on EOI and introduced the connected self as a mediating variable to explain the influencing mechanism. Thus, our study reveals the role of interpersonal processes in EOI construction and enriches the relevant literatures.

Finally, this study indicates the expansion of self-concept through the mediating role of connected self in EOI construction. Existing studies suggest that identity construction involves a process of depersonalization through which the positive features of organizations are incorporated into the collective self (Schmitt et al., 2006; Zhu et al., 2017). However, Curchod et al. (2014) revealed depersonalization in a system that did not take into consideration the individuality behind the organization. An employee’s self-concept is affected by action and context (Spears, 2001). Social identity theory also suggests that self-concept is fluid and the shift from personal to social identity represents an important type of fluidity in self-concept (Onorato and Turner, 2004). Consistent with these researches, our results indicate that relationship-building behaviors could transform an individual’s self-concept into a connected self. The construction of EOI means that an employee connects with others through relationship-building behaviors and expands his or her self-concept into a collective self. Compared with previous studies, our study focuses more on the process of self-concept expansion rather than depersonalization processes in construction of EOI. By introducing the connected self, our study reveals the process that employees engage in relationship-building behaviors to expand and fill their self-concepts in construction of EOI. Hence, this study provides new support for the expansion of self-concept and enriches the research on self-concept.

### Practical Implications

Our findings also provide some practical implications. In the era of VUCA, employees are full of uncertainty and anxiety, and organizations face a turbulent environment (Petriglieri et al., 2019). In order to ensure the connections between employees and organizations, organizations should take actions to promote EOI (Miscenko and Day, 2016), which is a stabilizing force that interrelates employees with their organizations (Ng, 2015). Based on the results of our study, organizations should appropriately change their management practices according to two perspectives to better promote the EOI.

Firstly, in terms of the relationship-building of EOI construction, organizations should consider what social resources should be put in place to help employees build positive relationships. This includes relevant people (e.g., colleagues, supervisors, and mentors), organizational activities (e.g., group activities), and social networks (e.g., contact lists for different types of knowledge, skills, and interests). Organizations can provide the facilities, spaces, and opportunities to make it easier for employees’ relationship-building behaviors to occur, such as free coffee, tea rooms, on-site gyms, and collective time, etc. Organizations should also create appropriate climate and provide relationship-building training programs to facilitate interpersonal behaviors. These management practices could increase relationship-building behaviors among employees and help them to construct EOI.

Secondly, in terms of the social comparison of EOI construction, organizations should stimulate employees’ positive social comparison behaviors to enhance the impact of relationship-building behaviors. Organizations can set positive role models and let their images widely spread so that employees could easily compare themselves to these prototypical employees. Organizations can create transparent organizational climate that provide chances and information for employees to make positive social comparisons. For example, an organization can establish the information sharing platform and online community to facilitate the flow of information about colleagues and the organization, which makes employees feel treated equally. Organizations can also hold work related events regularly to enhance positive social comparisons, such as experience exchanging meetings, professional knowledge and skills competitions, etc.

### Limitations and Future Directions

There are also several limitations in our study and some potential future directions for additional research. First, in terms of research methods, although we collected data in two phases, all variables were self-reported by the employees, which might result in common method variance. We followed several procedural techniques to minimize common method variance through the research design (Podsakoff et al., 2003), such as ensuring participants’ anonymity and emphasizing that there were no good or bad answers. Furthermore, we tested the common method variance using statistical control methods such as Harman’s single-factor test and the unmeasured latent method construct technique. We discovered that common method variance had no significant effects on our measurements. Future research should use the experimental method to collect data to reduce the effects of common method variance and better understand the actual causal relationships between the variables.

Second, in terms of research content, we assessed the role of interpersonal processes in construction of EOI, which is only one part of the identity construction process. Ashforth and Schinoff (2016) summarized that there are other processes in the process of individuals defining themselves in organizations. Our results cannot systematically reflect the complex situation of identity construction. In the future research, we can further explore other paths of the identity construction from different frameworks. Future research could consider both the cognitive and interpersonal processes of identity construction to reveal the mutual influence between individual cognition and interpersonal interaction in the construction of EOI. Based on this paper, future research can incorporate variables related to individual’s self-concept into the model to explore the process of shift from “I” to “We.” In addition, social comparison is divided into different dimensions, which have different influences on individuals (Li et al., 2021). Future research can also consider the influence of different dimensions of social comparison on EOI.

Third, in terms of data sources, we used an online survey platform (Credamo) to investigate samples from China, where social relationships play a more prominent role in individual identity construction due to the collectivist culture. Therefore, we controlled for the collectivism orientation of employees in our model. Future research might empirically test our research model using contrasting samples from individualistic countries to improve the generalizability of our research. Although Credamo that is similar to MTurk in terms of functionality has been used in previous literatures (Huang and Sengupta, 2020; Gai and Puntoni, 2021; Ma et al., 2021), future studies should collect data in the field to further ensure the authenticity and controllability of the data.

## Conclusion

In closing, our empirical evidence suggests that employees’ relationship-building behaviors promote their organizational identities by enhancing employees’ connected selves. In addition, the mediated relationship between relationship-building and EOI through the connected self is stronger when employees engaged in more social comparisons. Our findings extend knowledge regarding EOI construction as viewed through the lens of self-concept and reveal the role of interpersonal processes in the EOI construction. Considering the importance of relationship-building and social comparison in the process of identity construction, managers should not only create a platform and environment for employees to build relationships but also set an example for employees to make social comparisons, which might enable employees to construct more positive organizational identities.

## Data Availability Statement

The raw data supporting the conclusions of this article will be made available by the authors, without undue reservation.

## Author Contributions

GY, JS, and YS participated in the design of this study. JS performed the statistical analysis and drafted the manuscript. GY and YS provided comments on different versions of the manuscript. All authors contributed to the article and approved the submitted version.

## Conflict of Interest

The authors declare that the research was conducted in the absence of any commercial or financial relationships that could be construed as a potential conflict of interest.

## Publisher’s Note

All claims expressed in this article are solely those of the authors and do not necessarily represent those of their affiliated organizations, or those of the publisher, the editors and the reviewers. Any product that may be evaluated in this article, or claim that may be made by its manufacturer, is not guaranteed or endorsed by the publisher.

## Footnotes

^1^The higher the sample credit score we set during the questionnaire collection, the higher the quality of the questionnaire will be, but the speed of data collection is slower. Sixty points is the boundary value provided by the website, and the quality of the subjects above 60 points is qualified.
